# The history of 20^th^ century malaria control in Peru

**DOI:** 10.1186/1475-2875-12-303

**Published:** 2013-08-30

**Authors:** Sean M Griffing, Dionicia Gamboa, Venkatachalam Udhayakumar

**Affiliations:** 1 Division of Parasitic Diseases and Malaria, Malaria Branch, Center for Global Health, Centers for Disease Control and Prevention, Atlanta Georgia, USA; 2 Atlanta Research and Education Foundation, Atlanta Georgia, USA; 3 Departamento de Bioquimica, Instituto de Medicina Tropical “Alexander von Humboldt”, Universidad Peruana Cayetano Heredia, Lima, Peru; 4 Departamento de Ciencias Celulares y Moleculares, Facultad de Ciencias y Filosofia, Universidad Peruana Cayetano Heredia, Lima, Peru

**Keywords:** Latin America, Malaria, Peru, Public health, Plasmodium, Vivax, Falciparum, Disease, Malaria control, Malaria eradication, Drug resistance, Pesticide resistance, Habitat change

## Abstract

Malaria has been part of Peruvian life since at least the 1500s. While Peru gave the world quinine, one of the first treatments for malaria, its history is pockmarked with endemic malaria and occasional epidemics. In this review, major increases in Peruvian malaria incidence over the past hundred years are described, as well as the human factors that have facilitated these events, and concerted private and governmental efforts to control malaria. Political support for malaria control has varied and unexpected events like vector and parasite resistance have adversely impacted morbidity and mortality. Though the ready availability of novel insecticides like DDT and efficacious medications reduced malaria to very low levels for a decade after the post eradication era, malaria reemerged as an important modern day challenge to Peruvian public health. Its reemergence sparked collaboration between domestic and international partners towards the elimination of malaria in Peru.

## Early Peruvian malaria

Francisco Pizarro y González’s avaricious invasions of Peru during the 1500s likely spread malaria to the Incas. As a result, malaria would have predominated on the coastal plains, Andean foothills, and the interandean valleys. In the 1600s, Peru gave the world Cinchona tree bark, the source of quinine, which was the first effective anti-malarial used worldwide [1].

As the 20th century began, the Andes divided Peruvians between the Pacific coast and the Amazon. Both regions suffered childhood malaria exposure, leading to partial immunity (*Plasmodium vivax*, *Plasmodium falciparum*, and *Plasmodium malariae*). Andeans were immunologically naïve because vectors were absent above 1,500 m. The intelligentsia falsely assumed that Andeans had a racial weakness for malaria because outbreaks increased when they migrated to the coast. Such migration increased when plantations and associated businesses required workers during sowing and harvesting between March and August, which coincided with peak *Anopheles* densities due to increased breeding aided by seasonal rain and stagnant water accumulation. Andean workers had little access to preventative measures or treatment [2,3].

**Figure 1:**
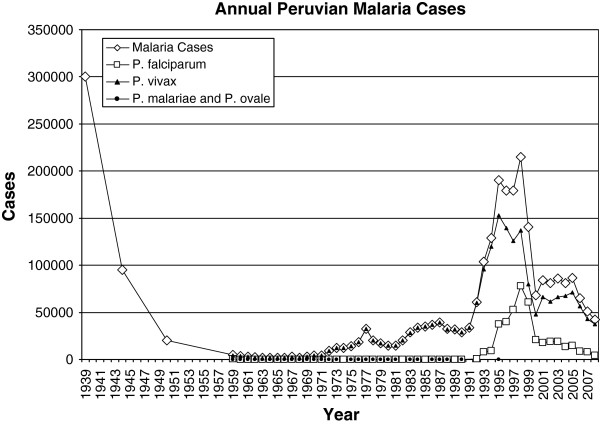
**This figure reports annual estimated malaria cases in total and by species, when available.** It also reports a number of significant events in Peruvian malaria control. Estimate quality likely varies based on multiple unmeasured factors. For example, the 1939 estimate may have occurred prior to suitable infrastructure. In addition, numbers reported for 1991–1994 and 1996–1999 were based on visual estimates from line and bar graphs and are reported for qualitative analysis [2,4-7].

Peruvian malaria prevalence has been influenced by pulses of immunologically naïve hosts confronting malaria for economic reasons, waxing and waning support for malaria control, and the loss of vector and parasite control measures (Figure 1).

### Early malaria control policy

As the 20^th^ century began, mosquito populations were controlled by mosquito nets, home fumigation, metal roofs, and draining or spraying water reservoirs with Paris Green or oil. The first national malaria control law came in 1916 after a coastal epidemic. Landowners were required to distribute quinine, destroy *Anopheles* larvae, and separate living quarters a minimum distance from rice paddies and sugar-cane fields. Quinine and metal roofing were no longer taxed. Some complied, maintained a medical service, provided quinine, and cleaned canals and ditches. They were unlikely to drain wetlands considered irrigation reservoirs or reconstruct housing. In the Amazon, a philanthropic landowner society managed a hospital from 1908 to 1918, with state support thereafter [2].

Compliance was inconsistent. In the 1930s, doctors and planters argued whether rice cultivation was associated with the patchy coastal malaria distribution. Doctors favored more separation of irrigated fields and worker living quarters, planters less. It was sometimes difficult to provide medical care because of sparse medical staff. Adequate treatment was limited by the belief that water spirits caused malaria. Patients were sometimes under dosed or overdosed with quinine and prescriptions sometimes disguised using unfamiliar names due to side effects [2].

A major Amazonian epidemic (likely *P. falciparum*) occurred during 1932 in Cuzco department, which was larger than local epidemics going back to at least 1898. Workers on the provincial outskirts were infected in August within the territory of the Machiguenga tribes. Recent deforestation, road building, and heavy rains led to puddles and stagnant water pools where mosquitoes bred. The epidemic spread rapidly along rivers and reached the Andean foothills, aided by the movement of merchants, workers, and fleeing citizens. The public health response was slow because Health Board was leaderless and the central government was disconnected from the Amazon. Staff unable to speak Quechua deployed amidst corruption accusations and adulterated quinine. The epidemic ran its course by May 1934*.* Out of a population of 25,000, there were 6,000-10,000 deaths and 15,000 sick. This led to the development of the Cuzco Health Directorate Anti-malarial Service [2].

Peru centralized its public health response in 1933 by creating the Health Directorate Hygiene and Prophylaxis Service, which purchased wholesale quinine from the National Agrarian Society. Initial efforts focused on malaria control in coastal Carabayllo. In 1937, another study was started in Cañete, south of Lima. These groups united to form the Malaria Expert Department of the recently created Public Health, Work, and Social Forecasting Ministry, which conducted malaria campaigns and constructed malaria hospitals. In 1939 the Health Ministry said malaria control was “meager, almost null” and that in certain regions it was “something that no one complained about or commiserated with; they accepted it as part of life.” Amazonian malaria control was thought impossible or not a priority. An observer said, “malaria will not disappear, while the waters are not channeled…while the swamps are not drained; all things that the doctors don’t know how to do” [2].

In 1941, the ministry created the National Rural Sanitation Antimalarial Service, which provided assistance, prevention, and statistics. Eight anti-malarial services were created in Lima, Cuzco, Ayacucho, Cañete, Chancay, Camaná, and the Moche and Tambo valleys. Road construction and public projects were supervised to disrupt larval breeding pools, hospitals were improved, and the supply of quinine was increased [2]. During the 1941 war with Ecuador, both armies suffered from malaria [8]. Due to yellow fever deaths in March and April, the Peruvian Director of Health asked the Rockefeller Foundation (RF) to take over yellow fever and malaria services under a cooperative agreement. The foundation expanded funding to the country and assigned a staff member [9].

In 1942, the Ministry became the Public Health and Social Assistance Ministry which increased the national services autonomy, staff, and budgets [2]. The Pan-American conference of 1942 led to the development of the Interamerican Public Health Cooperative Service (SCISP). SCISP employed 750 Peruvians and functioned until the 1970s with United States financial and technical support. In the early years, SCISP spent $1,350,000 dollars on hygiene education and an Amazon hospital, as well as public health projects in coastal Chimbote. Chimbote had been chosen for the first national steel works and fishery and it was thought this would encourage an influx of immunologically naïve people and another malaria epidemic. SCISP’s projects included draining seven swamps that covered 887,000 square meters, the installation of potable water systems, and the construction of a modern hospital [2]. The Pan American Bureau also contributed two staff members to Peru and had a close relationship with the country’s malaria services by 1944. The bureau contributed lab and field tests that led to the substitution of copper arsenite for Paris Green after WWII made it too expensive. They also provided assistance in the design and construction of the Peruvian copper arsenite factory [2,8,10]. Meanwhile, some argued that the Amazon would be habitable and profitable if public health was prioritized [2].

Between 1941 and 1948, the RF gave funds to Peru for various malaria efforts with support gradually decreased as government payments increased [2]. The RF’s initial malaria program goal was the eradication of *Anopheles pseudopunctipennis* from the Pacific Coast valleys with Paris Green starting with Lurín in 1943 [11]. The presence of virulent *Bartonella bacilliformis* in the upper valley kept non-immune staff from visiting; it was dangerous to stay overnight and impossible to make the round trip within daylight hours because it was inaccessible by car [12]. Other difficulties included drainage, seasonal changes in rainfall and water levels, and scarce labor [11]. Controlling the vector was inexpensive, but contemporary reports concluded eradicating it would require effort across the valley. Funds were increased four-fold and staff from the National Malaria Service received training, which shifted their focus from treatment to control [13]. The RF concluded that control was possible, but that eradication was not [2,14]. The RF also provided a survey and consultancy to the government regarding re-organizing the National Health Department, establishing local agencies, and advocating for full time staff. The department of Ica, which contained three of the fifty coastal valleys, was chosen for a model health service including malaria control. Later, another program was started in Arequipa [13,15]. The RF also contributed to the creation of the National Institute of Hygiene [15].

### The impact of DDT vector control

The RF introduced DDT to Peru in 1946. By 1947, the National Malaria Service was spraying DDT in 16 coastal valleys. By 1953, DDT was being sprayed in 47 coastal valleys and three in the Amazon. Malaria morbidity went from 945 per 100,000 (1941–1946) to 490 per 100,000 (1947–1958). In 1944, there were 95,349 malaria cases, but 20,000 by 1950. The coast accounted for 67% of cases that year (803 per 1,000) and in the Amazon 33% (295 per 1,000) [2]. After the 1950s control efforts, there were less than 1000 cases a year bordering Ecuador, Colombia, and Brazil, more than 80% of which were *P. vivax* and none *P. falciparum*[16].

By 1957, malaria control shifted to eradication through SCISP and UNICEF support. The country organized a Malaria Eradication National Service with funding from the National Health Fund and Social Welfare. UNICEF provided vehicles, equipment, insecticides and medications. SCISP contributed technical assistance, administration, and medication. It was projected that five years of aggressive anti-malarial and insecticides application would eradicate malaria and incidentally control yellow fever, dengue, and murine typhus [2].

The effort began in November 1957 with 67,633 DDT coastal sprayings. Malaria disappeared from some departments that same year. Between 1959 and 1962, sprayings were higher than 600,000 per year in five zones. By 1965, coastal Piura, Tumbes, La Libertad, Ica, Callao, Arequipa and Huancavelica had three years of interrupted transmission and there were only 1,500 cases countrywide [17]. By 1970, almost the entire coast was malaria free, as well as the interandean valleys, and the southern Peruvian Amazon [2,18]. Unfortunately, malaria increased again in 1970 as mosquitoes became DDT resistant and malaria control program funding decreased [2].

The success of malaria eradication, and an increasingly urbanized coast, encouraged Andean migration to the coast and Amazon. Chimbote went from 4,000 inhabitants in 1946 to 50,000 inhabitants by 1958. Still, between 1946 and 1955, malaria was the first ranked cause of Peruvian morbidity and the 10^th^ ranked for mortality. Malaria control efforts between 1940 and 1972 caused a social shift from a rural mountain population to a coastal urban one [2].

DDT use had halted in Loreto, an Amazonian department that constitutes one fourth of Peru’s area, in 1988, though bed nets were still used [17,19,20]. Between 1992 and 1997 malaria increased four-fold in Peru and fifty-fold in Loreto [17,19].

### Malaria reemergence after malaria eradication

Peruvian *P. vivax* cases gradually increased from 1,484 cases in 1963 to 39,122 cases in 1987 [4]. During the 1980s, Peruvian malaria control was unstructured [2]. A *P. malariae* outbreak occurred in Pampa Hermosa in the western Amazon, during 1986–1987, where before only *P. vivax* circulated [21]. In 1984, less than 1% of malaria cases were caused by Amazonian *P. falciparum*[22]. Three years later, there was a *P. falciparum* outbreak on the northern coast, no cases in 1988, and 65 in 1989 [4,20]. In 1990, there were 28,882 malaria cases, of which 131 were *P. falciparum*, principally in the northern coastal departments of Piura and Tumbes [4].

Malaria began to increase on the coast and along the periphery of Loreto. In 1991, Loreto had 140 *P. falciparum* cases with a Pastaza River valley outbreak, while a major outbreak began on the northern coast [17,20]. Cases were reported in the northern Loreto in Gueppi, to the west on the Pastaza River and in Yurimaguas, to east in Baja Putumayo and Atlántida, and to the southeast in Requena-Buenas Lomas [21]. In 1992, Pampa Hermosa reported its first *P. falciparum* cases. A year later, there were 436 *P. falciparum* cases, 205 *P. vivax* cases, and a few *P. malariae* cases [21]. Malaria was also reported in northwest Andean Cajamarca and *P. falciparum* incidence was increasing in the coastal departments [5,20].

*Plasmodium falciparum* was first reported in Padrecocha, a riverine village of 1,400 inhabitants near the major city of Iquitos, in 1994 [5,17]. Iquitos is the largest city in Loreto with a population of 305,514 in 1993 and 345,000 in 1999, while the remainder of Loreto had 474,000 inhabitants along the Amazon tributaries. It grew due to oil exploration, mining, and drug trafficking [17,19,23]. Andeans were encouraged to move to Iquitos due to government policy and fighting between the military and Shining Path during the 1980s and 1990s. This led to rural expansion and deforestation with an estimated 4,257 forest hectares cleared between 1983 and 1995. Another settlement extended away from Iquitos by following an unfinished road between Iquitos and Nauta, along with deforestation. Habitat changes including rice cultivation, irrigation canals, and aquaculture led to flooding that increased vector populations [19].

After the heavy rains and flooding caused by El Niño, conditions were ideal for the blooming of major epidemics [24]. By 1997, there were 121,268 slide confirmed malaria cases, of which 45% were *P. falciparum*, though *P. malariae* remained rare. Loreto accounted for 67.2% of malaria cases in Peru. While there were outbreaks throughout Loreto, most cases occurred around Iquitos in two high transmission areas, along the Nanay River downstream of Iquitos and along the Iquitos-Nauta road. Elsewhere in Loreto, the communities around the Yavarí and Pastaza Rivers had high transmission. Malaria was hypoendemic, with periods of mesoendemicity [17]. As the 1990s ended, the Amazon basin and the northern Pacific coast accounted for 85% of malaria cases and 95% of *P. falciparum* cases [5].

The Health Ministry organized a network of 329 health facilities and 850 promoters for observing Amazonian malaria to 1) diagnosis and treat malaria; 2) reduce disease severity and transmission; and 3) lower cost. Only 137 facilities could diagnose malaria by light microscopy, while the remainder collected blood smears. All facilities were authorized to provide treatment after smears were malaria verified. In 2001, there were 35,632 malaria cases in Loreto, of which 9,654 were *P. falciparum* with the main vector *Anopheles darlingi*[25]. Peruvian self-treatment was uncommon, despite pharmacies providing anti-malarials without prescription, because government treatment was free [5].

During 2004, malaria control was transferred to the National Health Strategy for the Prevention and Control of Tropical Diseases from the National Control Program, principally financed by national funds, but also USAID and the Global Fund to Fight AIDS, Tuberculosis and Malaria. By 2008, there were 1,292 health promoters and 293 microscopists. Domiciliary spraying with residual insecticides was a principal malaria control program, protecting 235,615 people. Almost 30,000 bed nets impregnated with insecticides were distributed in 2007. Rice cultivation was shifting from flooding during the crop growth phase to intermittent dry periods to control pyrethroid resistant vectors [6].

### Vectors

During the 1970s, 98% of the anopheline fauna was *Anopheles benarrochi,* where *P. falciparum* and *P. vivax* were endemic [18]. However, *Anopheles albimanus* may now be the primary malaria vector in coastal Peru [3]. Others argue it is *An. pseudopunctipennis*[26], the more efficient vector. Regardless, peak vector populations occur at the rainy season’s end [2].

In contrast, Amazonian rain disrupts *Anopheles* larval habitat. Greatest rainfall occurs between June and August. Rain is more persistent in the below sea level Amazon, limiting *Anopheles* breeding. During the Amazonian dry season, swamps and pools form where mosquitoes breed and spread malaria [19].

*Anopheles darlingi* was the major Peruvian Amazon vector prior to its elimination in the 1960s. Its reintroduction likely enhanced the epidemics of the late 1990s [17,19,27]. *Anopheles darlingi* was absent in 1991 in Iquitos, but present when *P. falciparum* infections rapidly increased. It made up more than 90% of the *Anopheles* around Iquitos during the rainy season, when most epidemic cases occurred, and remained the major dry season vector [27]. While *An. darlingi*’s reintroduction replaced *An. benarrochi* in the eastern and central Peruvian Amazon, *An. benarrochi* was the most important malaria vector in the western Amazon and limited eastern localities in the late 1990s [17,18,25,28].

### Drug resistance

As the 20th century began, malaria was treated with quinine and later plasmoquine and quinacrine [2]. In 1965, cases were chloroquine (CQ) sensitive on the Ecuadorian, Colombian, and Brazilian borders [17]. CQ resistance was reported on Putumayo River in the epidemics of Gueppi, Yubineto-Angusilla, and Alamo on the Colombian border in 1979 and 1980 [21,29]. CQ resistance was later reported in 1986 at Aquarico Rio, Alto Napo, and on the Pastaza river and its tributaries on the Ecuadorian border [21].

Amazon *in vivo* studies suggested that *P. falciparum* had reduced CQ and sulphadoxine-pyrimethamine (SP) sensitivity during 1993 to 1997. However, the Health Ministry did not consider them coordinated because of varying methods [5,30]. By the end of the 1990s, the National Malaria Program and Loreto Public Health Department provided malaria diagnosis and free CQ treatment as the first line medication, SP as the second, and quinine with clindamycin or tetracycline as the third for *Plasmodium falciparum* infection. Drug treatment varied by location, but CQ treatment was widely ineffective [17]. In 1996, SP replaced CQ as the first line treatment in central and eastern Amazon and was replaced by a seven day course of quinine plus tetracycline in 1997, based on limited *in vivo* efficacy data and drug resistance monitoring by the National Malaria Control Program [5].

The WHO recommended *in vivo* drug trials, which were conducted on the Pacific coast and the Amazon to assess the efficacy of CQ and SP. Caballococha in eastern Loreto and Iquitos had similar SP resistance in 1998 (56%, RII/RIII) [31]. In western Loreto, SP use was minimal because CQ had been the first line treatment until late 2002, though SP was occasionally used between 1997 and 1999 [5,24]. This was reflected in drug resistance; only 14.3% of patients had adequate clinical and parasitological response to CQ, but 92.3% had adequate clinical and parasitological response to SP in 2000. Molecular findings agreed [25]. In a 1999 study, coastal parasites had RII and RIII resistance to CQ between 53% and 65%, but RII and RIII resistance to SP varied from 0 to 10% [24]. SP and SP plus artesunate (AS) treatment efficacies were found to be 97% and 99% respectively for *P. falciparum*[3]. In Iquitos, mefloquine (MQ) and MQ plus AS had 97% and 99% efficacy for *P. falciparum* treatment [5].

Peru was one of the first countries in the Americas to adopt artemisinin-based combination therapy (ACT) for *P. falciparum* infection after its implementation in Southeast Asia. This decision was reached at a national malaria treatment policy meeting in Lima in 2000 after extensive clinical trials throughout Peru with support from USAID, CDC, and the US Naval Medical Laboratory [5]. The coast would use AS plus SP and the Amazon, AS plus MQ [5,32]. New first, second, and third line drug treatments were approved in August, 2000, while pregnant women continued to be treated with quinine plus clindamycin [5]. AS plus MQ therapy was implemented in and around Iquitos in 2001. In the northwestern Amazon, CQ was used for the first line treatment for *P. falciparum* in 2002. By January 2003, AS plus MQ was used in this region as well [25]. In Iquitos, changing drug policies decreased SP resistance, though CQ resistance remained constant. Molecular studies showed that the removal of SP drug pressure resulted in the decline of highly resistant parasites [23,33]. The Health Ministry shifted from generic to commercial drugs in 1999 [5]. This decision increased the cost of anti-malarials, though there was a reduction in time lost to disability in the Amazon [34].

The national treatment strategy for *P. vivax* infections was three days of CQ and seven days of primaquine (PQ), but compliance after the first three days was poor with a recrudescence risk [35]. For example, along the Iquitos-Nauta road, only 62.2% of patients completed their CQ and primaquine *P. vivax* treatment due to local beliefs [36]. As of 2007, pregnant women were treated with CQ during pregnancy and with PQ after [37]. There were also limited reports of CQ-resistant *P. vivax*[38].

### *Plasmodium falciparum* population dynamics

Microsatellite markers and mutations associated with drug resistance have been used to analyze Peruvian *P. falciparum* and *P. vivax* populations in Peru and give insight into population structure and response to medications [17,23,33,35,39-44]. There were at least five major clonal *P. falciparum* lineages present in Peru based on isolates collected between 1998 and 2000. It was hypothesized that there were at least two CQ and SP resistant clonal lineages that had spread from Amazonian Brazil, two SP sensitive clonal lineages that were descendants of parasites from the coastal Peru, Ecuador, or Colombia and had spread to the Peruvian interior, and a vestigial Peruvian Amazon interior clonal lineage [45]. Regarding *P. vivax*, it appears there is a lack of outbreeding, few polyclonal infections, and high linkage disequilibrium [35].

### Diagnostic-resistant *Plasmodium falciparum* parasites

Where microscopy cannot be used as the primary diagnostic tool, rapid diagnostic tests (RDTs) are being considered as an alternate tool in Peru. Most commercial malaria RDTs use histidine rich protein 2 (HRP2) as the target antigen, which is only presented by *P. falciparum*. In Peru, up to 40% of *P. falciparum* parasites had HRP2 deletions [46]. Such parasites generate false negatives for HRP2-based RDTs and therefore non-HRP2 based RDTs must be considered.

## Conclusions

Peru should be applauded for the success of its malaria control programs. It has learned to mitigate the migration of naïve hosts to malarious regions, provide adequate medical intervention, and monitor parasites for drug resistance. Malaria control programs in other countries should plan for the migration of naïve hosts to economic centers, maintain political will even as malaria dwindles, and prepare for the failure of primary anti-malarials and insecticides. Armed with this knowledge and techniques, Peru can control and possibly eliminate malaria.

## Competing interests

The authors declare no competing interests.

## Authors’ contributions

SG conceived this review, conducted the compilation of background material, and drafted the manuscript. VU supported the review and contributed to the manuscript design and critical revisions. DG contributed to the manuscript through additional references, revisions, and manuscript suggestions. All authors read and approved the final manuscript.
